# The quantitative investigation of spark plasma on skin parameters with skin elasticity, thickness, density, and biometric characteristics

**DOI:** 10.1038/s41598-023-34425-z

**Published:** 2023-05-12

**Authors:** Erfan Ghasemi, Mohammad Ali Nilforoushzadeh, Mohammadreza Khani, Mohammad Amir Amirkhani, Maryam Nouri, Parisa Charipoor, Mohammad Eftekhari, Samira Izadpanah, Babak Shokri

**Affiliations:** 1 Laser and Plasma Research Institute, Shahid Beheshti University, G.C., P.O. Box, Tehran, 19839-6941 Iran; 2 Skin and Stem Cell Research Center, Tehran University of Medical Sciences, Tehran, Iran; 3 Skin Repair Research Center, Jordan Dermatology and Hair Transplantation Center, Tehran, Iran; 4 Physics Department of Shahid, Beheshti University, G.C., P.O. Box, Tehran, 19839-6941 Iran

**Keywords:** Plasma physics, Medical research

## Abstract

Cold atmospheric plasma has been developed and utilized as a novel technique for skin rejuvenation because of its various effects on cells and living things. This study investigated the accuracy of this claim and any possible side effects of using spark plasma to rejuvenate skin. The present work is the first quantitative investigation using animal models. 12 Wistar rats were divided into two groups for this investigation. To compare the skin's natural process with the treated skin, the first group underwent a single session of plasma therapy, while the second group served as the control group. The back of the necks of the samples was shaved for 20 cm. Before beginning treatment, the MPA9 multifunctional skin tester was used to determine the melanin index, erythema index, and transepidermal water loss (TEWL). The skin's thickness and density were assessed using sonography, and its elasticity index was calculated using a Cutometer. The samples were exposed to plasma radiation in the designated area (in a triangular pattern). The abovementioned signs were examined immediately after the following therapy and at the weekly appointment 2–4 weeks later. Optical spectroscopy was also used to demonstrate the presence of active species. In this study, we found that a plasma spark therapy session significantly boosts skin elasticity, and the ultrasound results revealed a significantly increased skin thickness and density. The plasma increased the amount of skin surface evaporation, erythema, and melanin immediately following the treatment. However, 4 weeks later, it recovered to its former state and did not differ significantly from before the therapy.

## Introduction

People from all walks of life want to live a healthy lifestyle and appear younger in the twenty-first century as the global population ages^1^. Aging is a natural process that affects all of the body's systems^2^. Skin aging is influenced by internal factors like chronological age and external factors like smoking and prolonged UV exposure. Several characteristics, including skin firmness, elasticity, and moisture loss, are connected to skin aging. Loss of elasticity is the first sign of skin aging, so maintaining elasticity should be the first step in skin aging treatment^3,4^.

The variety of modern skin care options includes both physical and chemical treatments. While licensed beauticians frequently provide skin peeling treatments, creams, serums, and oils are commonly utilized as at-home cosmetic treatments. Although some equipment can be purchased for personal use, beauty salons still provide physical therapies ^1^. LED lights and lasers, for example, are frequently used for rejuvenation. By physically destroying the skin's outer layers (regeneration), these light sources stimulate skin renewal by turning on the skin's cellular metabolism ^5,6^.

Although ablative lasers have been used successfully in the last decade to reduce many symptoms of damaged skin, erythema, itching, and acne-like pimples are all transient side effects of lasers. Non-ablative lasers, on the other hand, have few side effects but are less effective in clinical settings^5,6^.

An optimum treatment should be chosen based on the patient's goals and preoperative analysis (normalization of tissue and pigment alterations, increase in skin collagen, and atrophic soft tissue volume) to maximize clinical effectiveness in a short recovery period while minimizing significant side effects^9,10^. On the other hand, The need to overcome the limitations and difficulties posed by lasers has led to the search for alternative methods and tools that may employ mechanisms other than laser technology. One approach proposed to achieve this goal is plasma skin regeneration (PSR), which uses plasma to transfer energy. Hadian et al.^10^. Cold plasma is as effective and safe as long-pulsed Nd: YAG laser treatment but with less discomfort and dryness. Potter et al. ^8^. Found that six months after the plasma treatment, fine lines and wrinkles were reduced by 24%. The "fourth state of matter," plasma, is produced when ionizing neutral atmospheric gases ^7^. The atmospheric gas between the electrode of the medical device and the skin is ionized to produce plasma energy. This ionized gas contains ions, electrons, active oxygen species (OH, H2O2, …), active nitrogen species (NO, NO2, …), an electric field, heat, and other elements^8,9^.

Cold plasma has enormous potential in the medical field^10,11^. The use of cold atmospheric pressure plasma (CAP) in surgery and the treatment of cancer is just one example of its many medical uses^8,10–12^. Additionally, it works well for skin care^12,13^, sterilization^11^, and dentistry^7,14^. Today's entry of CAP into the cosmetics industry creates a new challenge. CAPs can be a promising alternative in non-invasive skin treatment due to their unique ability to produce a complex chemical composition and physical properties^1^.

In numerous studies, it has been demonstrated that using cold atmospheric plasma (CAP) improves the oxygenation of the underlying tissue^1,9^. Increased temperature (between 30 and 40 °C) and NO produced directly or indirectly by CAP may be to blame for this^9^.

This technique may be utilized in various energy conditions to achieve multiple effects, ranging from superficial epidermal effects comparable to microdermabrasion to deeper skin heating akin to carbon dioxide^15^.

The spark medical devices generate plasma energy by ionizing atmospheric gas between the device and the skin. The resulting plasma spark sublimates the surface layers, instantly transfers the stored heat energy to the skin surface, and uniformly heats it ^26,27^. Plasma radiofrequency (P-RF) energy generates microplasma sparks in the air between the device's tip and the skin's surface, causing mild epidermal erosion and superficial dermal perforation with a 1 mm diameter spot. Mild epidermal sublimation preserves the epidermis and prevents damage to the deeper layers of the skin. In addition to a mechanical effect that shapes the surface on which it impacts, the detached spots of the sublimation technique induce a thermal effect that promotes skin regeneration and extensive dermal fibroblast remodeling, including new collagen synthesis and deposition, while also stimulating rapid re-epithelialization^28^. Heat shocks can cause an increase in procollagen type I and procollagen type II expression^13^. As a result, they can stimulate cells to produce more collagen.

This study aimed to investigate the quantitative effects of Spark plasma on skin parameters such as skin firmness and elasticity, thickness and density of skin layers, melanin index, erythema index, stratum corneum hydration, and epidermal water loss. Using skin analyzer devices like Cutometer, Skin ultrasonography, Tewameter, and Mexameter in treated areas provides the ideal circumstances for boosting attractiveness in patients seeking skin rejuvenation. All the factors listed above were quantitatively and statistically studied to establish the benefits and drawbacks of utilizing this procedure in skin rejuvenation.

## Result

### Visual assessment of plasma performance

**Figure 1:**
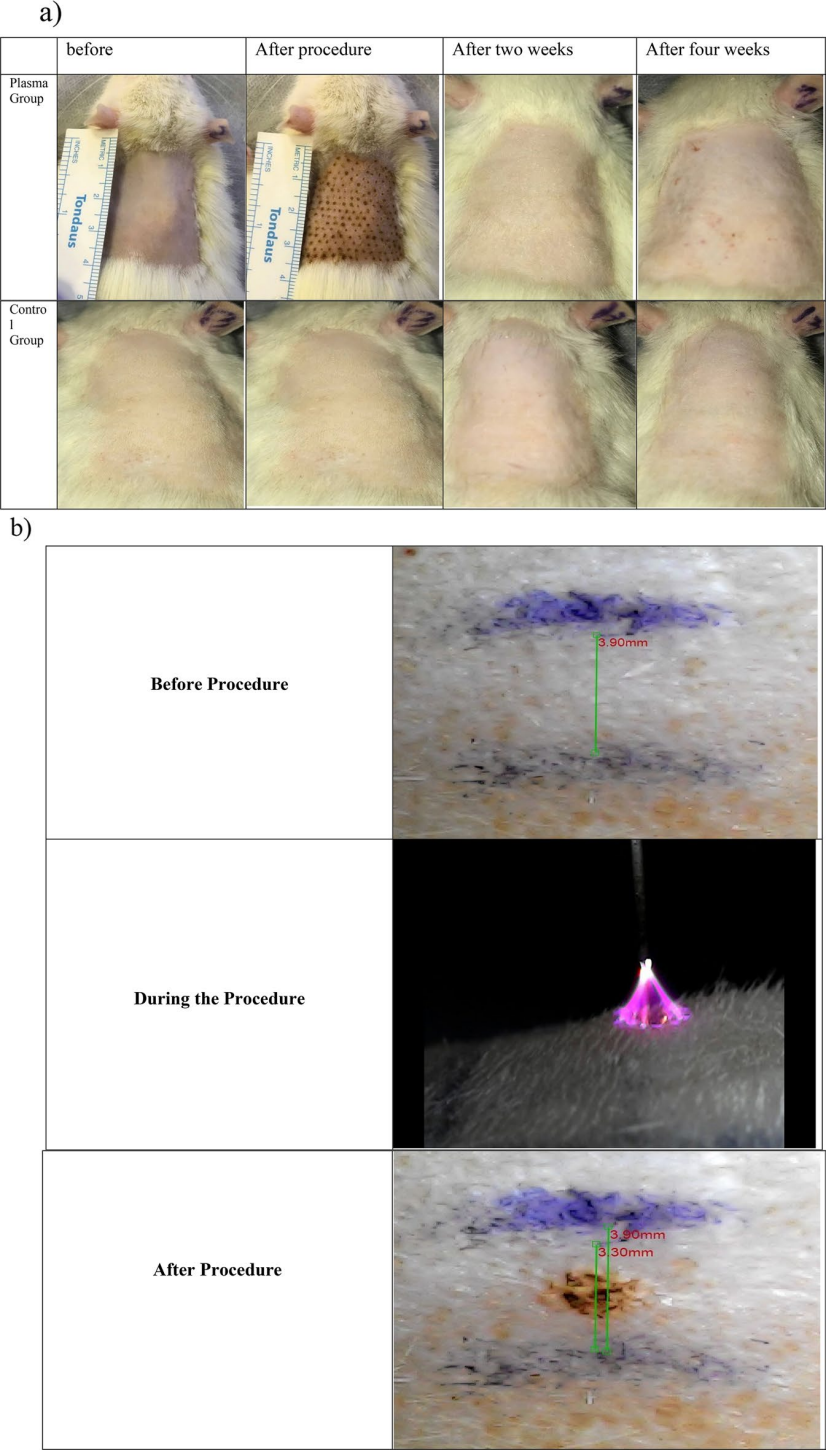
Visual evaluation of plasma performance in rats (**a**) over 4 weeks. (**b**) Skin shrinkage rate immediately after treatment.

Figure 1.a depicts the evolution of the plasma process over 4 weeks. The first row is about plasma processing, and the second represents the control group. The skin changed noticeably after therapy, as Fig. 1a illustrates. The immediate plasma effect, which caused the skin to contract at the processing site, was considerable in animal samples. It was decided to use quantitative tests to evaluate effectiveness for a more accurate assessment. It is worth noting that the burned areas vanished after two weeks, leaving no visible scars. Shortening the skin's superficial hair causes small red spots. Within two weeks, all minor and transient side effects had subsided. Figure 1b also shows that the skin shrank from 3.90 to 3.30 mm (15.38% reduction) in the plasma mode that was meant for treatment, demonstrating that skin lifting and wrinkle removal are both possible with the application of plasma spark^16,17^.

### Effects of plasma on biometric parameters

#### Cutometer

**Figure 2:**
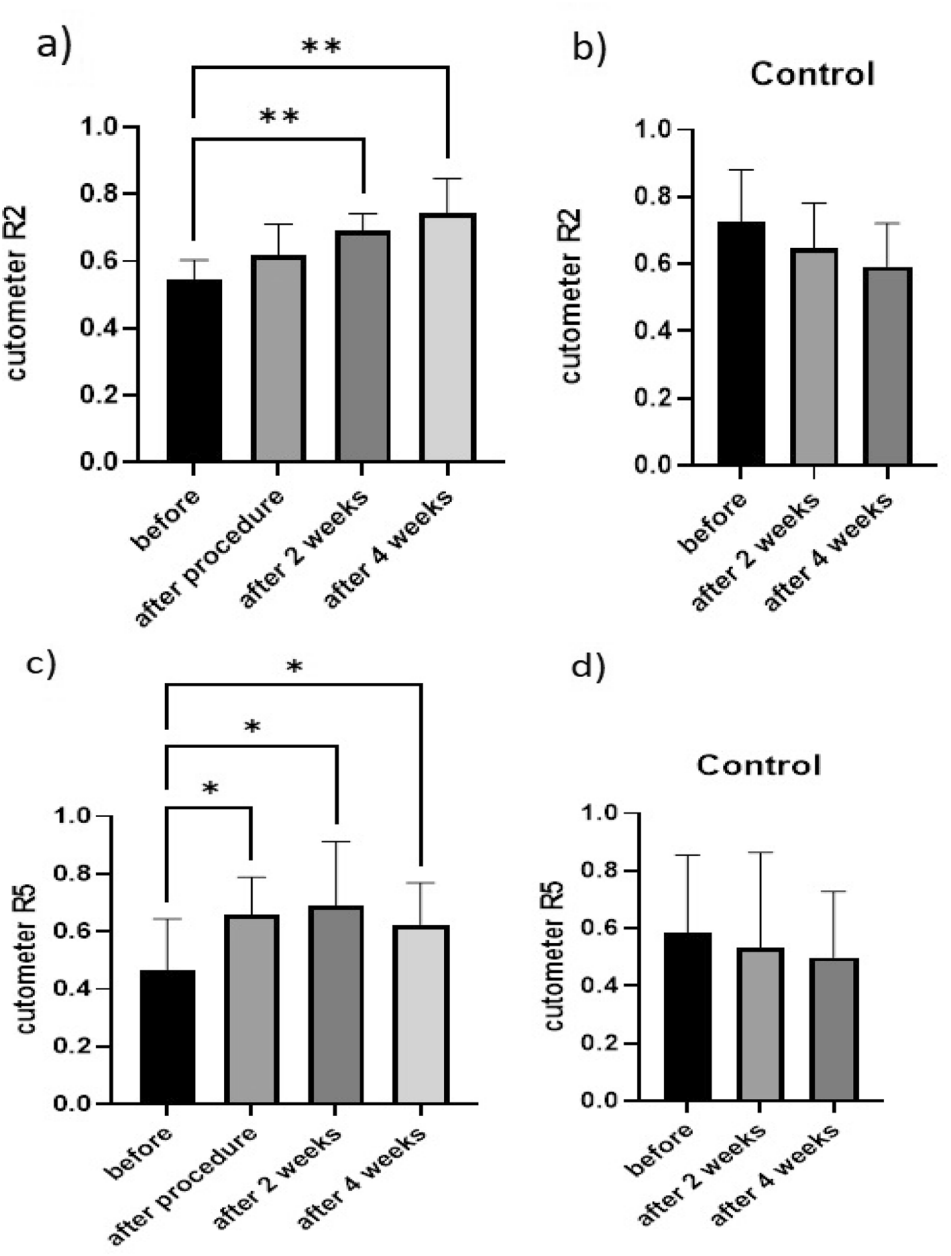
Comparison of skin elasticity parameters in rats after receiving plasma and a control sample over 4 weeks (**a**) R2 parameter of the plasma group, (**b**) R2 parameter of the control group, (**c**) R5 parameter of the plasma group, (**d**) R5 parameter of the control group. [*p < 0.05, **p < 0.01, a significant difference from before the experiment began].

According to Fig. 2a, the average value of the R2 parameter, which represents resistance to mechanical force versus recovery ability, was 0.5467 before processing but increased to 0.6173 immediately after, most likely due to skin shrinkage. After two weeks, it increased significantly compared to before treatment and reached a value of 0.6929, and finally, in the fourth week of follow-up, it maintained this linear growth. It got a value of 0.7458 compared to before treatment. This growth pattern indicates that skin tolerance has increased following plasma treatment.

In contrast, the average R2 value in the control group was 0.7265 at the start of the experiment. Still, it fell to 0.6454 by the second week of follow-up and to 0.5930 by the fourth week. Figure 2b reveals that the control group's average R2 value was not significantly different.

Furthermore, as shown in Fig. 2c, the average value of R5, representing the elastic part of the suction phase versus the immediate recovery in the relaxation phase, gradually increased after one session of plasma processing in the treatment group. The mean value has risen from 0.4627 to 0.6596 immediately after treatment. It increased significantly in the second week of follow-up, reaching an average of 0.6890. Furthermore, it maintained a significant increase in the fourth week of follow-up, reaching an average of 0.6235. Figure 2a and c provide additional evidence supporting the hypothesis that plasma does not harm skin elasticity but increases it.

However, the average R5 parameter value in the control group was 0.5842 before the experiment's commencement, 0.5307 during the second week of follow-up, and finally, 0.4986 during the fourth, as shown in Figure 2b, d, indicating that there was no significant difference in the control group.

#### Skin ultrasonography

Table 1 shows that the treatment group's average skin density dropped from 13.46 to 10.68 immediately following plasma treatment. However, following the completion of the skin repair procedure, the average skin density rose to 14.54 in the second week of follow-up and then significantly to 19.71 in the fourth week. The skin ultrasound results revealed denser layers in the dermis and epidermis, indicating that plasma affects skin density. The average densities of the epidermis and dermis were 42.08 and 7.878 percent, respectively, before plasma processing. Following processing, these values dropped to 32.29 and 4.973 percent, respectively, but during the second week's follow-up, the average densities of the epidermis and dermis rose to 38.74 and 9.437 percent, respectively. Finally, they achieved an average density of 52.22 and 13.1 percent in the fourth week of follow-up, a significant improvement from the baseline. In the control group, however, the average skin density at the start of the experiment was 14.44 percent. This value decreased to the baseline level in the second week of follow-up and reached an average of 12.11, but it nearly returned to the baseline level in the fourth week (Table 1).

**Table 1:** Comparison of rat skin thickness and density during four weeks: group treated with plasma, the control group.

Groups	Plasma						Control				
Measurement time	Before	After procedure	After 2 weeks	After 4 weeks	p value	The percentage of changes (%)	Before	After 2 weeks	After 4 weeks	p value	The percentage of changes (%)
Skin density (%)	13.46	10.68	14.54	19.71	0.0389 p < 0.05	46.43	14.44	12.11	14.29	0.9045 p > 0.05	− 1.03
Skin thickness (µm)	810.5	801	880.3	1019	0.0361 p < 0.05	25.72	882.6	908.2	852.6	0.6695 p > 0.05	− 3.39
Epidermis density (%)	42.08	32.29	38.74	52.22	0.0428 p < 0.05	24.09	47.58	38.94	46.24	0.6963 p > 0.05	− 2.81
Epidermis thickness (µm)	135.6	142	150.6	172	0.0258 p < 0.05	26.84	148	151.5	145.7	0.7028 p > 0.05	− 1.55
Dermis density (%)	7.878	4.973	9.437	13.1	0.0427 p < 0.05	66.28	7.69	6.705	7.98	0.8053 p > 0.05	3.77
Dermis thickness (µm)	672.6	648.6	743.8	884.2	0.0392 p < 0.05	31.46	736	781.8	694.5	0.5037 p > 0.05	− 5.63

[p value (before, after four weeks), The percentage of changes from the commencement of the tests to the fourth week of follow-up]].

Table 1 also shows a significant increase in average skin thickness. The average skin thickness of the treatment group decreased from 810.5 to 801 µm immediately after plasma treatment. Table 1 shows that immediately following treatment, plasma changed skin thickness at the treatment site by 9.5 µm. However, following the completion of the skin repair process, the mean skin thickness increased to 880.3 µm in the second week of follow-up and then increased dramatically to 1019 µm in the fourth week of follow-up.

**Figure 3:**
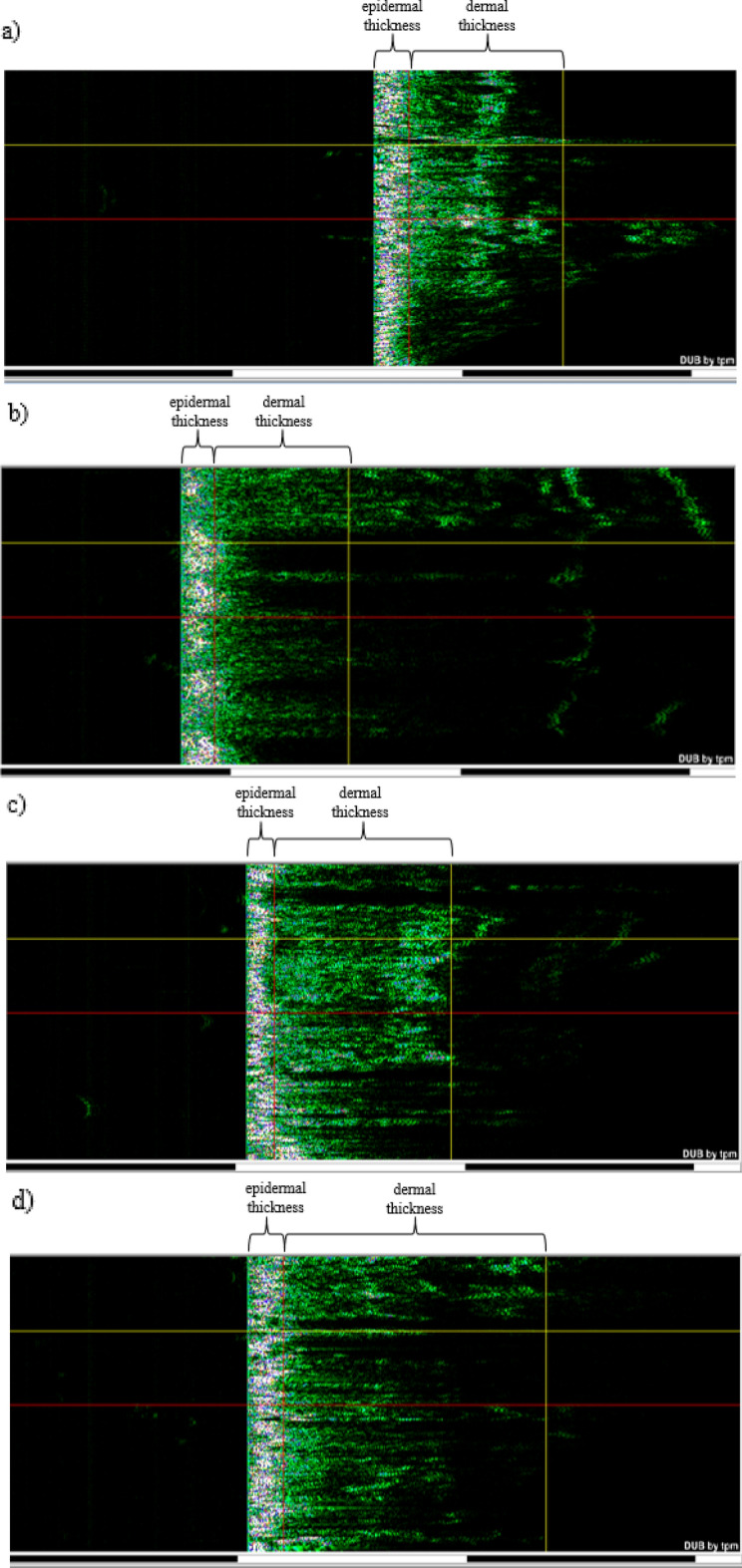

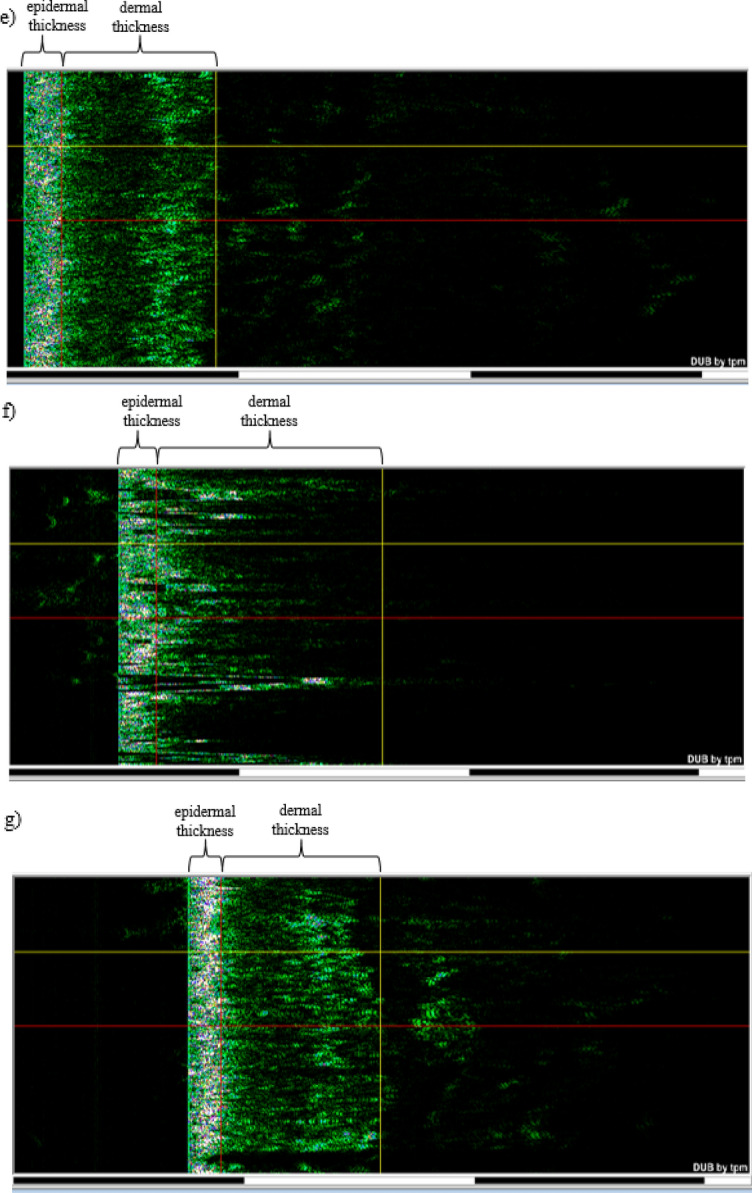
Skin thickness and density were obtained using ultrasound. Dermis is displayed from a red to a yellow vertical line, and the epidermis is indicated from a green to a red vertical line. Plasma group: (**a**) before, (**b**) immediately after treatment, (**c**) 2 weeks later, (**d**) 4 weeks later. Control group: (**e**) before the experiment, (**f**) after 2 weeks, (**g**) after 4 weeks.

As shown in Table 1, the average thickness of the epidermis and dermis decreased initially after plasma treatment. Still, it increased significantly in the fourth week of follow-up compared to the baseline. According to Table 1, the average skin thickness in the control group was 882.5 µm before the experiment began, 908.2 µm in the second week, and 852.6 µm in the fourth week, indicating no significant difference in skin thickness in the control group. Figure 3 also shows ultrasound images of a sample from the treatment group and a sample from the control group, which demonstrate the changes in dermis and epidermis thickness during the research.

Ye et al. ^37^ demonstrated that the thickness and density of the skin change as a consequence of burns. Since Table 1 supports this finding, the difference in the density and thickness of the skin after treatment is most likely due to heat transfer and partial burns.

The simultaneous analysis of Fig. 2 and Table 1 supports the assertion that plasma can be one of the most effective techniques for skin rejuvenation because plasma significantly increases skin elasticity by significant thickening and densifying the skin. In contrast, there was no discernible difference in the control group.

#### Tewameter

**Figure 4:**
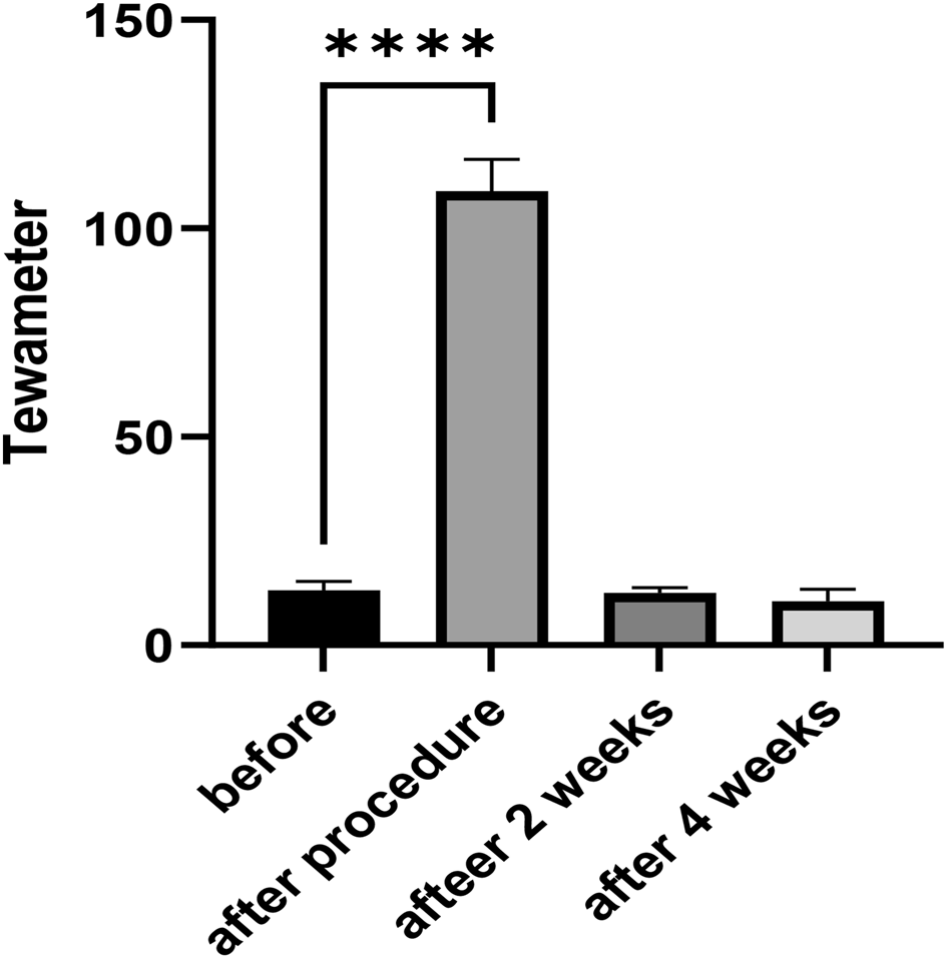
Changes in mouse skin's Tewameter parameter over four weeks following plasma processing [****p < 0.01, a significant difference from before the experiment began].

Figure 4 demonstrates that the skin surface of the treatment group saw a significant increase in evaporation rate (g/h/m^2^) due to heat transfer to the skin, indicating that the skin has lost its barrier effect. The average rate of evaporation increased from 13.22 to 109. However, it decreased and reached 12.62 in the second week of follow-up, indicating that the skin repair process is most likely complete. The mean finally fell below the baseline in the fourth week of the study, recording a value of 10.62. This demonstrates that after 4 weeks, plasma has no effect on the skin barrier and has no drying effect on the skin ^18^. Additionally, it can be said that it complies with the fundamental guidelines for protecting human skin.

**Figure 5:**
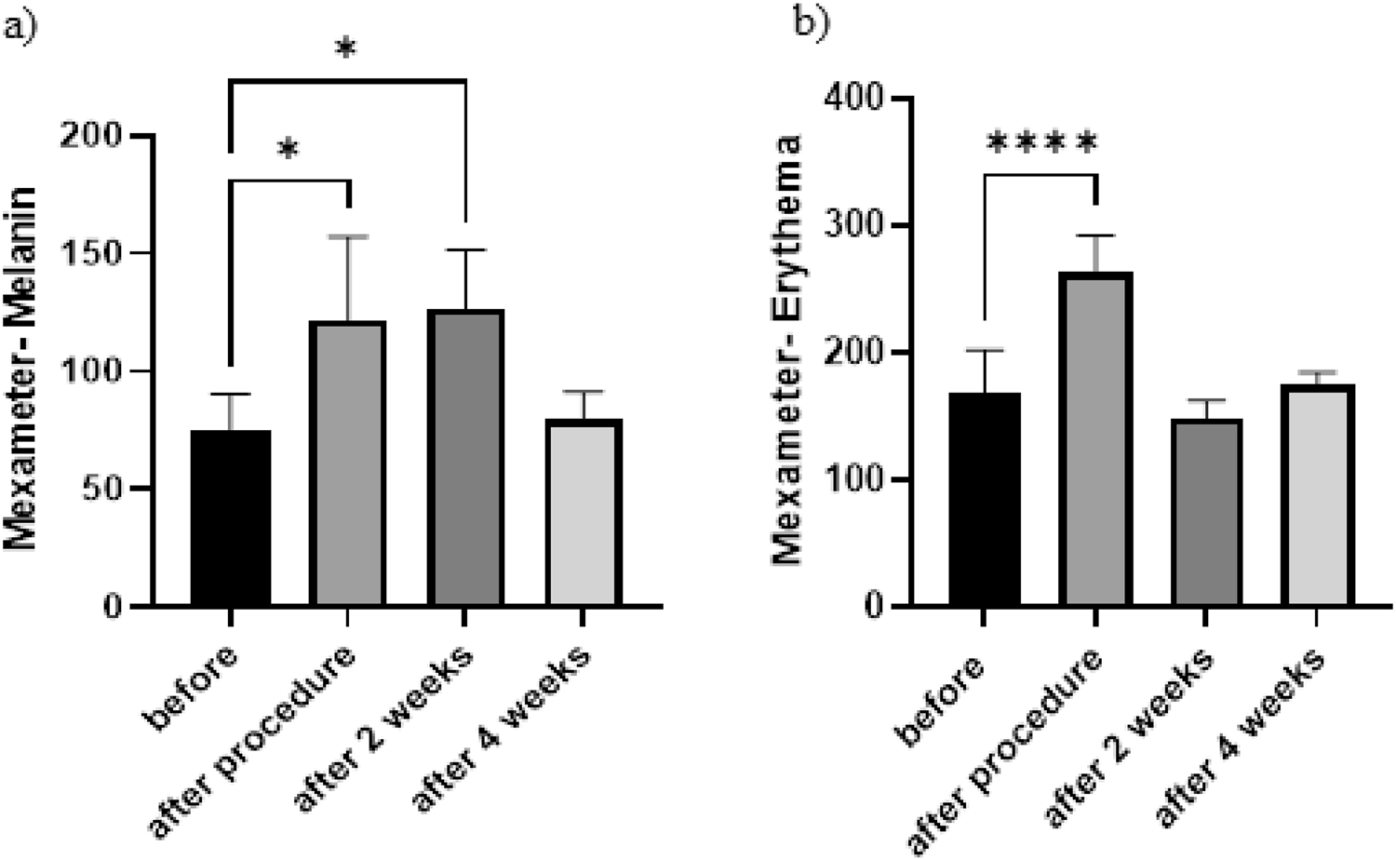
Comparison of the mouse skin mexameter parameters over four weeks following plasma processing: (**a**) Melanin; (**b**) Erythema [*p < 0.05, ****p < 0.0001, a significant difference from before the experiment began].

Mexameter. Figure 5a shows that in the following treatment, melanin levels and the number of skin pigments significantly increased from 75.28 to 121.5, most likely due to black soot from minor skin burns (Fig. 2a). The average melanin dropped to 79.67 in the fourth week of follow-up, which was not statistically different from before the beginning of treatment. Also, according to Fig. 5b, the amount of erythema on the skin in the treatment group increased significantly from 167.5 to 262.9 immediately after treatment, indicating inflammation. Still, in the second week of follow-up, the average decreased to 148.3, indicating that the inflammation had subsided. Finally, four weeks later, it increased to a mean of 174.9, with no significant change from pre-treatment levels. This theory, which holds that the plasma need not interact with the chromophore^9,19^, is supported by Fig. 5.

## Discussion

research indicates that NO has a significant impact on the skin, particularly on the growth of keratinocytes and fibroblasts^9,20,21^. Cold plasma can cause epidermis thickening, most likely due to keratinocyte proliferation^9,20^. This could be due to active nitrogen and reactive oxygen species effects on specific cytokines and subsequent cell growth in the treated areas^9,22^. Suschek discovered that the vasodilator effect of NO in plasma could increase skin microcirculation without causing any side effects ^22^. As skin blood flow increases, inflammatory cells invade, synthesize various growth factors and cytokines, and stimulate cell proliferation, including fibroblasts. Furthermore, NO can promote the synthesis of collagen IV and activate endothelial cell adhesion^1,23^. In both in vitro and in vivo models, Duchenne et al. ^24^ demonstrated that CAP treatment stimulates endogenous NO synthesis.

After plasma exposure, the human stratum corneum observed a transient in vivo water loss ^25^. The non-dried epidermis protects thermally damaged layers during recovery, a desirable effect of plasma skin resurfacing^26^. Because CAPs can deposit charges on the treated surface, the skin may absorb more water molecules after plasma treatment. In the first seconds of plasma treatment, the wettability of the human stratum corneum increases rapidly^27^. Increased hydrophilicity was also observed in fingernails, where plasma treatment improved nail polish adhesion^28^.

Furthermore, the electrical current transmitted by CAP, which penetrates the layers of the skin, can be beneficial. Since the early twentieth century, high-frequency electrotherapy has been used to treat various skin and other diseases^29^. A specific electrical stimulation has been shown to accelerate wound healing by increasing dermal fibroblast motility. Furthermore, direct and pulsed currents have been shown to stimulate keratinocyte differentiation, epidermal proliferation, vascularization, and the formation of new collagen^30–37^. As a result, many cold plasma-related physical factors can impact cellular activity and the microenvironment of the skin, either separately or in combination^1^. All of this emphasizes the beneficial role of CAP in skin biology and offers appropriate justifications in favor of using cold plasma to restore the skin's functional barrier and thereby enhance its health. Besides, the plasma is not required to interact with the chromophore^9,19^. The skin's epidermis is made up of two kinds of cells: keratinocytes, which are abundant in all layers of the epidermis and generate keratin to protect the epithelial cells from mechanical and non-mechanical stresses, and melanocytes, which are abundant in the epidermis's basal layer and create the protective pigment melanin^38^. It was discovered in this study that 4 weeks after plasma treatment, there is no significant difference in skin melanin and erythema, which is consistent with the finding that plasma does not require interaction with the chromophore. As a result, it produces more consistent results than ablative lasers^9,26^. Pigmentation alteration is one of the most prevalent side effects of laser resurfacing. It is frequently transitory, appearing within three weeks and lasting for a year or less^6^. In cases treated with CO_2_ lasers, hyperpigmentation can reach up to 46%^32^. Though less common (4 percent of cases treated with Er: YAG), hypopigmentation is a significantly more robust and potentially permanent side effect of laser therapy on all skin types^6^. According to Costa et al., fractional lasers can be harmful. Such side effects are characterized as either recent (occurring during the first seven days of treatment) or late (occurring around the second week)^33^. This study observed no pigment alterations in the final follow-up phase. This could be because plasma does not have to interact with chromophores.

One of the key advantages of this technology over fragmented lasers that act uniformly on the entire treated region is the ability to customize treatment millimeter by millimeter. Furthermore, the lack of adverse effects associated with the lack of a light source and the plasma's ability to adjust heat transmission and lessen the inflammatory impact promotes good cutaneous stimulation^34^. This method has several advantages, including the absence of absolute contraindications, minimal pain during surgery, rapid healing, rapid formation of a protective layer after surgery, rapid healing of the wound surface, immediate return to regular activity, and optimal results in increasing skin elasticity. Finally, the low cost of equipment compared to laser devices provides value in favor of the operator and the patient. They now have access to effective treatment at a substantially lower cost than laser treatment.

## Conclusion

Maintaining elasticity should be the first step in skin aging treatment because the loss of elasticity is the earliest indicator of skin aging ^3,4^. Finally, this paper proved that Plasma Spark could improve skin thickness, density, and hence skin elasticity, most likely due to increased keratinocytes and fibroblasts ^9,13^.

The mean TEWL increased immediately after treatment, indicating that the plasma accelerated the rate of water evaporation from the skin surface; however, after 4 weeks, the skin had recovered, and the mean TEWL was lower than the original value, demonstrating that the plasma does not affect TEWL.

Additionally, the average erythema increased immediately after the treatment, which may have been caused by heat transfer to the skin, decreased after two weeks, and the skin showed no overt effects in the fourth week. In conclusion, plasma spark is a novel and affordable method to rejuvenate the skin without compromising the skin barrier, which can be inferred from this article.

## Materials and methods

### Animal models and research groups

Male Wistar rats (4 months old) weighing 250 + 50 gr acquired from the Pasteur Institute (Tehran, Iran) were utilized in this experiment. Twelve rats were divided into two groups and kept in separate cages under conventional laboratory settings (room temperature, atmospheric pressure, humidity 30 + 10%, light/dark cycle 12 h) with easy access to water and food. After the experiment, all the mice were donated to Shahid Beheshti University's Faculty of Biology for survival.

### Animal ethics and ARRIVE guidelines

Animal experiments were conducted according to the Guidelines for Animal Care and Use Committee of Tehran University of Medical Sciences. All animal experiments described were approved by the Animal Experimentation Ethics Committee of Tehran University of Medical Sciences, IRAN (IR.TUMS.MEDICINE.REC.1400.766). The in-vivo study is reported in accordance with the ARRIVE guidelines (Animal Research: Reporting of In Vivo Experiments) for reporting experiments ^39,40^.

### Plasma device

**Figure 6:**
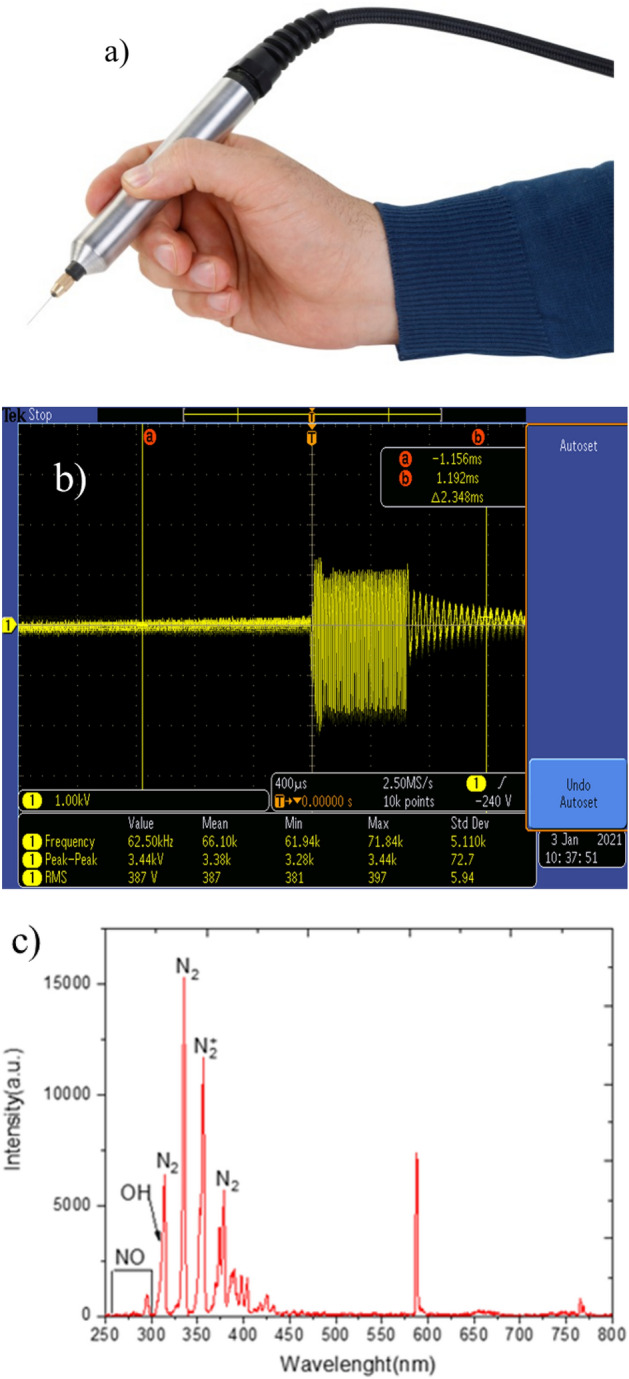
Cold plasma device characteristics. (**a**) Plasma device, (**b**) voltage and frequency, (**c**) the optical emission spectrum of the plasma device and the excited species produced by it.

The present work used the spark plasma system from Plasma Fanavar Jam company (Plasma Beuty 100- Fig. 6a). The electrical properties of the plasma device were tested and demonstrated using an oscilloscope and a high-voltage probe (Tekterorix, P6015A, 1:1000). The voltage changes are sinusoidal, as illustrated in Fig. 6b. The device has a peak-to-peak voltage of 3.44 kV and a frequency of 62.5 kHz. Optical emission spectrometry (OES; Avaspec3648USB2) was used to investigate various active species of cold plasma species. The OES spectra were collected in the 250–800 nm range along the plasma axis. The optical emission spectroscopy of the plasma device revealed the emission of species such as OH (309 nm), NO (297 nm), and N2/N2+ (315, 337, 358, 375.4, and 380 nm) in Fig. 6c^9,35^.

### Biometrics evaluation

The biometric characteristics of the samples, including melanin index, erythema index, stratum corneum hydration, and epidermal water loss, were measured using a multifunctional skin tester (Courage + Khazaka Electronics, Cologne, Germany). The elasticity index was computed using a Dual Cutometer® Dual MPA 580 Courage + Khazaka electronic GmbH (Köln. GERMANY).

The cutometer shows the skin's resistance to negative pressure (stiffness) and its ability to return to its original position (elasticity) as a curve (penetration depth in millimeters/time) in real-time during the measurement. We examined the tensile index using the metrics R2 and R5, R2 is the viscoelasticity in percentage, which reflects resistance to the mechanical force versus the ability of recovery, and R5 is the net elasticity in percentage, which represents the elastic part of the suction phase vs. immediate recovery during the relaxation phase^4,36^.

A skin ultrasonic imaging device (Dub®SkinScanner Taberna pro medium) (Luneburg, GERMANY) was used to measure the thickness of skin layers. This device allows for the visualization of structures up to a maximum depth of 1 cm^36^. A probe with a frequency of 75 MHz was used in this experiment. Skin samples were biometrically analyzed before and immediately after treatment at 2 and 4 weeks. All measurements were taken while relaxing in a controlled physical environment (room temperature 23 °C and humidity 40%).

### Treatment and preparation

12 Wistar rats were divided into two groups of six to assess the efficacy of Spark plasma and its potential effects on skin rejuvenation. The first group received plasma, while the second was a control group. Rats were anesthetized with ketamine hydrochloride (100 mg/kg) and xylazine hydrochloride (10 mg/kg) before plasma treatment. The area behind the rats' necks was cut short using scissors, then entirely shaved and measured with a ruler. The mouse's skin was cleansed, and a topical Xyla-P cream was applied to provide local anesthesia before irradiation. After 20 min, the area was cleaned and treated in a triangular pattern using a plasma device. After treatment, the area of the treated region was measured again using a ruler, and the amount of skin shrinkage was also calculated. To report the potential side effects of this method and compare it to the samples' normal skin process, skin analysis and biometrics were performed before and immediately after treatment, as well as at 2 and 4 weeks.

### Statistical analysis

Results were expressed as mean ± standard error of the mean (mean ± SEM). Statistical data analysis was performed by applying way-ANOVA to compare the groups using the Graph Pad Prism (9.0.0) software. The significance level was considered less than 0.05 (p < 0.05).

## Data Availability

The datasets used and analyzed during the current study are available from the corresponding author upon reasonable request.
